# Supplementary material for: An Integrative ATAC-Seq and RNA-Seq Analysis of the Endometrial Tissues of Meishan and Duroc Pigs
Source: Int J Mol Sci. 2023 Sep 30;24(19):14812. doi: 10.3390/ijms241914812 (PMC10573446; doi:10.3390/ijms241914812)
Supplement: Supplementary file 1 [file ijms-24-14812-s001.zip › ijms-2589584-supplementary.pdf]

Table S1. Quality control for raw and clean data in ATAC-seq.

Sample	Raw reads	Clean reads	Clean ratio	Q30-raw	Q30-clean
DR1	122235606	119044166	94.89%	83.13%	89.62%
DR2	132686038	127212552	95.87%	85.54%	94.57%
DR3	109107538	103012118	94.41%	83.76%	91.38%
MS1	130846692	122505518	93.63%	79.11%	86.46%
MS2	117326036	111493444	95.03%	82.14%	89.43%
MS3	122235606	116457536	95.27%	86.05%	92.57%

Table S2. Results of reference genome mapping and peak calling.

Sample	Mapped (million)	Mapping rate	Proper pairs	Peaks
DR1	82.4	95.4%	93.8%	6322
DR2	82.4	95.8%	94.6%	15921
DR3	71.6	96.9%	95.4%	49050
MS1	88.7	97.2%	95.1%	66285
MS2	72.0	95.6%	94.1%	60588

MS3	79.2	95.9%	94.4%	33973

Table S6. Statistical summary of the quality of RNA-seq data.

Sample	DR1	DR2	DR3	MS1	MS2	MS3
Raw Bases	7,142,567,100	7,235,890,500	7,357,030,200	6,241,110,300	6,129,940,500	6,938,005,800
Clean Bases	6,882,907,200	6,773,370,900	7,063,044,600	6,024,828,000	5,922,114,900	6,624,875,100
Low-quality Reads	241,114	242,024	194,916	122,430	147,636	155,194
Low-quality Read Rate (%)	0.51	0.5	0.4	0.29	0.36	0.34
Ns Reads	208,250	561,046	214,958	109,182	112,608	141,710
Ns Read Rate (%)	0.44	1.16	0.44	0.26	0.27	0.31
Adapter Polluted Reads	1,281,702	2,280,394	1,550,030	1,210,270	1,125,260	1,790,634
Adapter Polluted Read Rate (%)	2.69	4.73	3.16	2.91	2.75	3.87
Raw Q30 Base Rate (%)	90.12	89.79	90.16	92.59	92.35	91.93
Clean Q30 Base Rate (%)	90.41	90.27	90.41	92.77	92.55	92.13

Table S7. Distribution of mapped reads on the pig genome.

Library	DR1	DR2	DR3	MS1	MS2	MS3

Total Reads	45,886,048	45,155,806	47,086,964	40,165,520	39,480,766	44,165,834
Mapped Reads	43,519,369	42,952,415	44,743,187	38,027,727	37,222,904	40,683,728
Mapping Rate	0.9484	0.9512	0.9502	0.9468	0.9428	0.9212
UnMapped Reads	2,366,679	2,203,391	2,343,777	2,137,793	2,257,862	3,482,106
MultiMap Reads	3,618,939	1,182,401	4,612,973	1,061,790	1,158,014	1,183,266
MultiMap Rate	0.0789	0.0262	0.098	0.0264	0.0293	0.0268

Table S10. Information on primers used in this study

Primer	Sequence (5′–3′)	Annealing temperature	Product size
GAPDH-F	GGGCATGAACCATGAGAAGT	58°C	229 bp
GAPDH-R	AAGCAGGGATGATGTTCTGG		
FOXA2-F	TCCCACAGGTCGCTTGCT	60° C	154 bp
FOXA2-R	TTGCGGCTCGCTCAGATT		
SOX17-F	GGAGGAGCGGAGCAAATC	62°C	169 bp
SOX17-R	GGGCAACTGTGGGAAACC		
WNT6-F	TACCAGCATCTGCAGGAAGG	60°C	204 bp

WNT6-R	GTCTCCCGGATGTCCTGCT		
ADCY6-F	GACCATTGGGAGCACGTACA	58°C	177 bp
ADCY6-R	CCCATGTTCAGCCCGATCTT		
RIMKLB-F	CCTGTTGGCAGCAGAGAAAT	58°C	111 bp
RIMKLB-R	CATTTTTGCCCCTCCTGCTTG		
NOP2-F	CAGTCGTGCTCGAAAGAGGG	58°C	121 bp
NOP2-R	TGTCTGGACGGCTCCTTTTG		
DMRT1-F	TCCTCTGGATAGGGTGTC	58°C	206 bp
DMRT1-R	TCTACTTGAATAACTTGGGTC		
BRINP2-F	ACAACTCTGATTCAACCCTT	58°C	196 bp
BRINP2-R	CCCTGGCAACATACTACC		
DLX3-F	CAATCCAGGTGCTTTCCG	58°C	224 bp
DLX3-R	TTTGCTGCGTCCATTTCTC		
ANXA4-F	GCTTGGGCACTGATGACAAC	58°C	136 bp
ANXA4-R	CCGATGTGTCACCCTTGATG		
